# DiscoverY: a classifier for identifying Y chromosome sequences in male assemblies

**DOI:** 10.1186/s12864-019-5996-3

**Published:** 2019-08-09

**Authors:** Samarth Rangavittal, Natasha Stopa, Marta Tomaszkiewicz, Kristoffer Sahlin, Kateryna D. Makova, Paul Medvedev

**Affiliations:** 1 Department of Biology, Pennsylvania State University, University Park, PA 16802 USA; 2 Department of Computer Science and Engineering, Pennsylvania State University, University Park, PA 16802 USA; 3 The Genome Sciences Institute of the Huck Institutes of the Life Sciences, Pennsylvania State University, University Park, PA 16802 USA; 4 Department of Biochemistry and Molecular Biology, Pennsylvania State University, University Park, PA 16802 USA

**Keywords:** Genome assembly, Y chromosome, Male genome

## Abstract

**Background:**

Although the Y chromosome plays an important role in male sex determination and fertility, it is currently understudied due to its haploid and repetitive nature. Methods to isolate Y-specific contigs from a whole-genome assembly broadly fall into two categories. The first involves retrieving Y-contigs using proportion sharing with a female, but such a strategy is prone to false positives in the absence of a high-quality, complete female reference. A second strategy uses the ratio of depth of coverage from male and female reads to select Y-contigs, but such a method requires high-depth sequencing of a female and cannot utilize existing female references.

**Results:**

We develop a *k*-mer based method called DiscoverY, which combines proportion sharing with female with depth of coverage from male reads to classify contigs as Y-chromosomal. We evaluate the performance of DiscoverY on human and gorilla genomes, across different sequencing platforms including Illumina, 10X, and PacBio. In the cases where the male and female data are of high quality, DiscoverY has a high precision and recall and outperforms existing methods. For cases when a high quality female reference is not available, we quantify the effect of using draft reference or even just raw sequencing reads from a female.

**Conclusion:**

DiscoverY is an effective method to isolate Y-specific contigs from a whole-genome assembly. However, regions homologous to the X chromosome remain difficult to detect.

**Electronic supplementary material:**

The online version of this article (10.1186/s12864-019-5996-3) contains supplementary material, which is available to authorized users.

## Background

The mammalian Y chromosome plays an important role in male sex determination and fertility by harboring the *SRY* gene [1] and housing multi-copy genes [2, 3]. However, most de novo genome sequencing projects focus on the female reference, with less than one in five mammalian reference genomes having their Y chromosome sequenced [4]. This disparity is explained by difficulties associated with assembling the Y chromosomes due to its haploidy (resulting in low coverage) and highly repetitive nature.

The human Y was the first published Y chromosome, and provided an insight into the organization of mammalian Y chromosomes in general [5]. It includes regions unique to the Y chromosome; for instance, the ampliconic regions are organized into palindromes and contain many genes with a high copy number. Other regions, such as the X-degenerate regions, have regions with differing degree of homology to the X chromosome and contain single-copy genes. Pseudoautosomal regions (PAR) are those that have high homology to, and continue to recombine with, the corresponding regions on the X. The X-transposed region, which is unique to the human, is a recent transposition from the X that shares a high homology. Additionally, the human Y is enriched in heterochromatic regions.

The earliest methods for assembly of Y chromosomes involved a laborious technique of Single-Haplotype Iterative Mapping and Sequencing (SHIMS), which relies on the use of mapped large-insert clones (usually bacterial artificial chromosomes) derived from a single haplotype so that polymorphisms do not confound the assembly of ampliconic repeats, thus providing a high-quality reference at the cost of time and expense [5–8]. With the ubiquity of high-throughput sequencing, enrichment-based methods have been shown to produce draft-quality assemblies of the Y chromosome that successfully capture all Y-specific genes using a fraction of the resources required by the SHIMS method [9]. In one such method, Y-specific DNA is first enriched by an experimental technique such as flow sorting [9]. From the resulting set of all sequencing reads, Y-specific reads are isolated by leveraging their high abundance in the data set due to enrichment [10]. Such methods have been demonstrated to be effective in retrieving the Y chromosome of great apes such as the gorilla [9] and human [11]. However, to avoid artifacts associated with DNA amplification and cell propagation, they require a large amount of starting DNA material, which may not be available (e.g. for endangered species); they also require specialized wet-lab techniques which may not be widely accessible.

The alternative to enrichment-based techniques is to first do standard whole-genome sequencing and assembly of a male and second to isolate male-specific contigs computationally after assembly. There are two broad classes of such methods. The first strategy, represented by the Y-Genome Scan (YGS) method, uses comparison to a female reference to isolate Y-linked contigs [12]. Those contigs which have a low proportion of constituent *k*-mers shared with a female are shortlisted as Y-specific. This method has been demonstrated to work on human and fruit fly genomes [12]. However, such methods are sensitive to the quality and completeness of the female reference.

The second strategy, represented by the Chromosome Quotient (CQ) method, computes coverage information from reads from a sequenced male (*male reads*) and reads from a sequenced female (*female reads*) [13]. Those contigs, which have a significantly greater number of male reads mapping to them (as compared to female reads), are shortlisted as Y-specific. This method has been demonstrated to work on different avian, reptile and insect sex chromosomes [13–15]. However, such methods do not take advantage of available female reference genomes.

To combine the advantages of both these approaches, we propose a method called DiscoverY that takes contigs from the whole-genome sequencing and assembly of a male and classifies which of the contigs come from the Y, also using information from a female genome. It uses two features: the proportion of sequence shared with female and the depth of coverage from male reads. We demonstrate the ability of DiscoverY to retrieve Y contigs and study how various aspects of the sequencing datasets affect its accuracy. We test DiscoverY on both human and gorilla genomes, using Illumina, 10X, and PacBio SMRT technologies. DiscoverY is open source and freely available at [16].

## Results

### Human datasets

Table 1 summarizes all the datasets that we used for evaluating DiscoverY and Additional file 1: Table S1 provides the citation and download information for all the datasets. For the male, we used three assemblies of the same human (NA24385), generated from Illumina, 10X, and PacBio (i.e. Single-Molecule Real Time) data, respectively. These assemblies were pre-processed as described in the Methods section. We also varied the female data that we used. The first was the female reference obtained by removing the Y chromosome from the high-quality hg38 reference. While this is likely to give the best results, it is unlikely that such a high-quality reference would be available for a non-model or even a non-human organism. Therefore, we also experimented with female references generated from 10X and PacBio data for the NA12878 female. Note that we used male and female individuals that come from different ethnicities (Ashkenazim male and Utah female), in order to more accurately reflect what might occur in a real DiscoverY use-case (i.e. when sequencing non-human samples). To experiment with the case when a female reference is not available, we used Illumina reads for the same female. Full details of all these datasets are provided in Table 1 and Additional file 1: Table S1.

**Table 1:** Datasets used to test DiscoverY

Dataset				Reads			Assembly			
Species	Sex	Tech nology	Sample	Read length	Read number	Autosome coverage depth	Contig number	Contig N50	Total length (gb)	Y^1^ length (mb)
human	M	Illumina	NA24385	250 bp	883 mil	60x	65,436	169 kb	2.84	18
human	M	10X	NA24385	151 bp	477 mil	20x	359,515	24 kb	7.71	24
human	M	PacBio	NA24385	10kb^2^	13 mil	30x	12,523	4.5 mb	2.99	18
human	F	Illumina	NA12878	148 bp	5.5 bil	300x	N/A			
human	F	mixed	hg38 - Y	N/A			23	156.0 mb	3.03	N/A
human	F	10X	NA12878	N/A			21,562	16.2 mb	2.85	N/A
human	F	PacBio	NA12878	N/A			18,903	26.8 mb	3.17	N/A
gorilla	M	Illumina	see Methods	150 bp	406 mil	20x	N/A			
gorilla	M	N/A	gorGor5 + gorY	N/A			16,329	9.6 mb	3.10	25
gorilla	F	Illumina	Gg6	150 bp	141 mil	7x	N/A			

^1^Length of contigs aligning to the reference Y chromosome. For gorilla male, this is known directly from the construction, rather than through alignment

^2^Read N50 is shown instead of length, since the length varies

### Best-case performance of DiscoverY

**Fig. 1:**
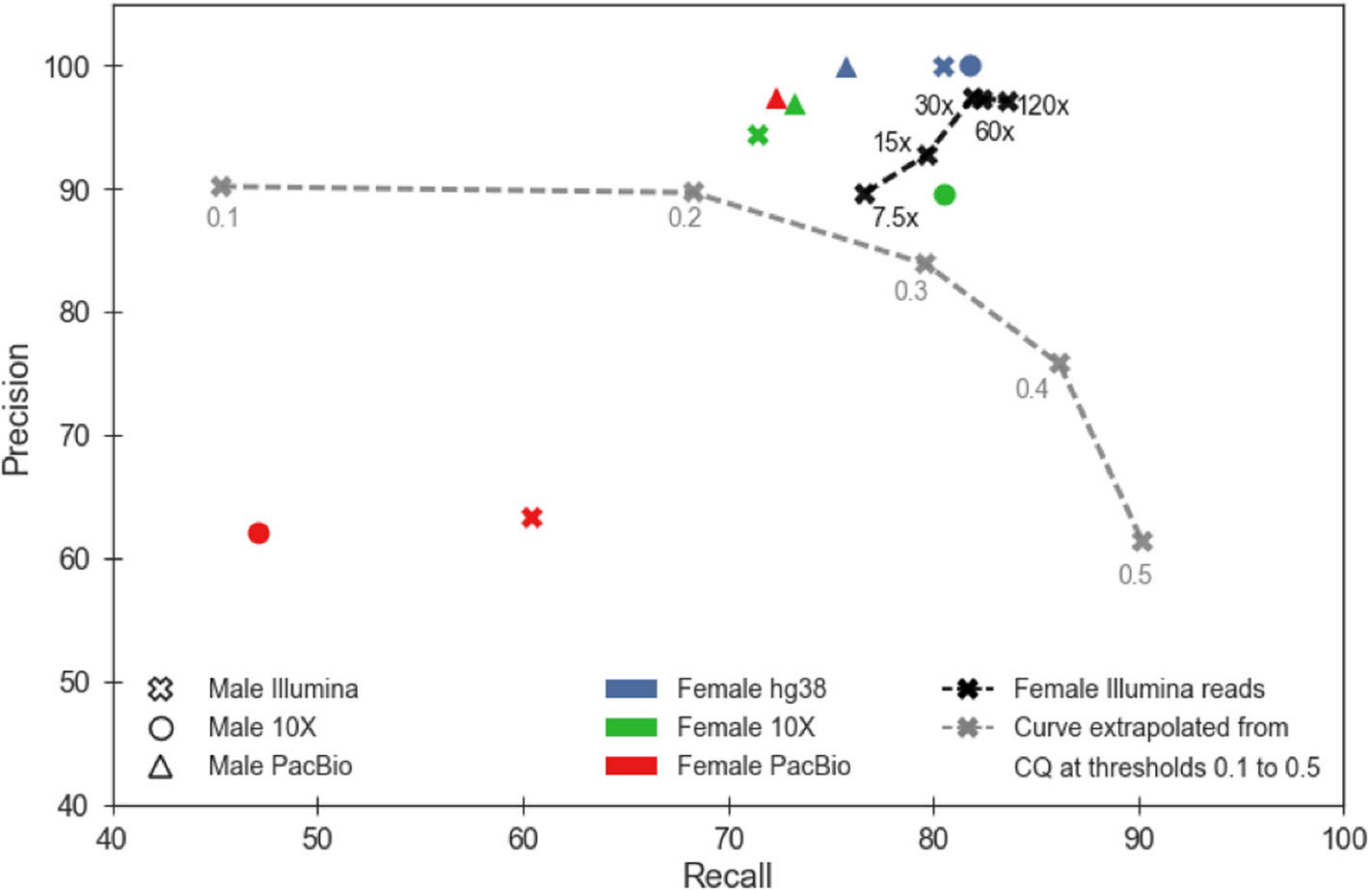
Precision and recall of DiscoverY on human data. Each marker represents the result of a DiscoverY experiment. The shape of the marker represents the male assembly used, and color of each marker represents the female reference used. The black, x-shaped markers connected by a dashed line show the performance of DiscoverY with female raw reads (instead of a female reference) at various levels of autosomal coverage. The CQ method is shown as a grey curve extrapolated from running CQ with thresholds 0.1 to 0.5 (in increments of 0.1)

Figure 1 shows the various precision and recall while varying the male and female datasets (Additional file 1: Table S3 contains the raw numbers). DiscoverY was run in female+male mode with optimal parameters, in order to understand the potential power of the method; the effect of parameter choices is instead explored in Section 3.4. Broadly, DiscoverY maintains precision of > 90% and recall of > 70% across most experiments.

**Fig. 2:**
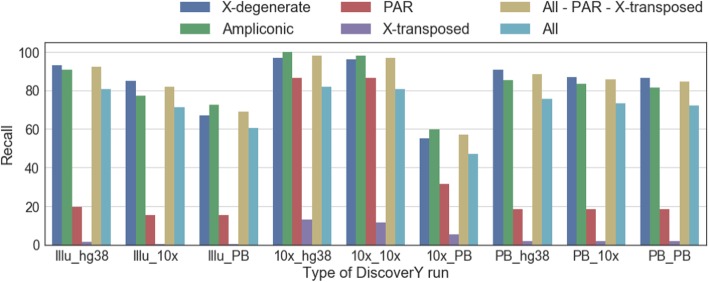
Recall of DiscoverY, broken down by region of the Y chromosome. The datasets are denoted on the x-axis, e.g. Illu_hg38 means the Illumina male dataset and the hg38-based female reference. The brown bar corresponds to recall on all the Y regions excluding the PAR and the X-transposed region. The recall is measured as the fraction of contigs coming from the region (as determined by alignment) that are labeled as Y by DiscoverY

We first explore how well DiscoverY can perform when high-quality male and female data are available. The best accuracy was obtained with the 10X male and hg38 female, with a precision > 99.99% and recall of 82%; we focus on this assembly in this section. Figure 2 breaks down the recall by region of the Y chromosome. The most challenging is the X-transposed region, where < 15% is recovered. The PAR region was also challenging to benchmark, because almost all of the PAR maps to the X chromosome, a majority of the PAR (> 99%) is filtered out during alignment while selecting the “True Y for Mapping”. The very small amount of PAR that escapes mapping can be correctly classified by DiscoverY, with recall of 85%. Besides these two regions, however, the recall was > 95%, which also includes ampliconic regions. Most, but not all, X-degenerate genes were recovered by DiscoverY (Additional file 1: Figure S1).

**Fig. 3:**
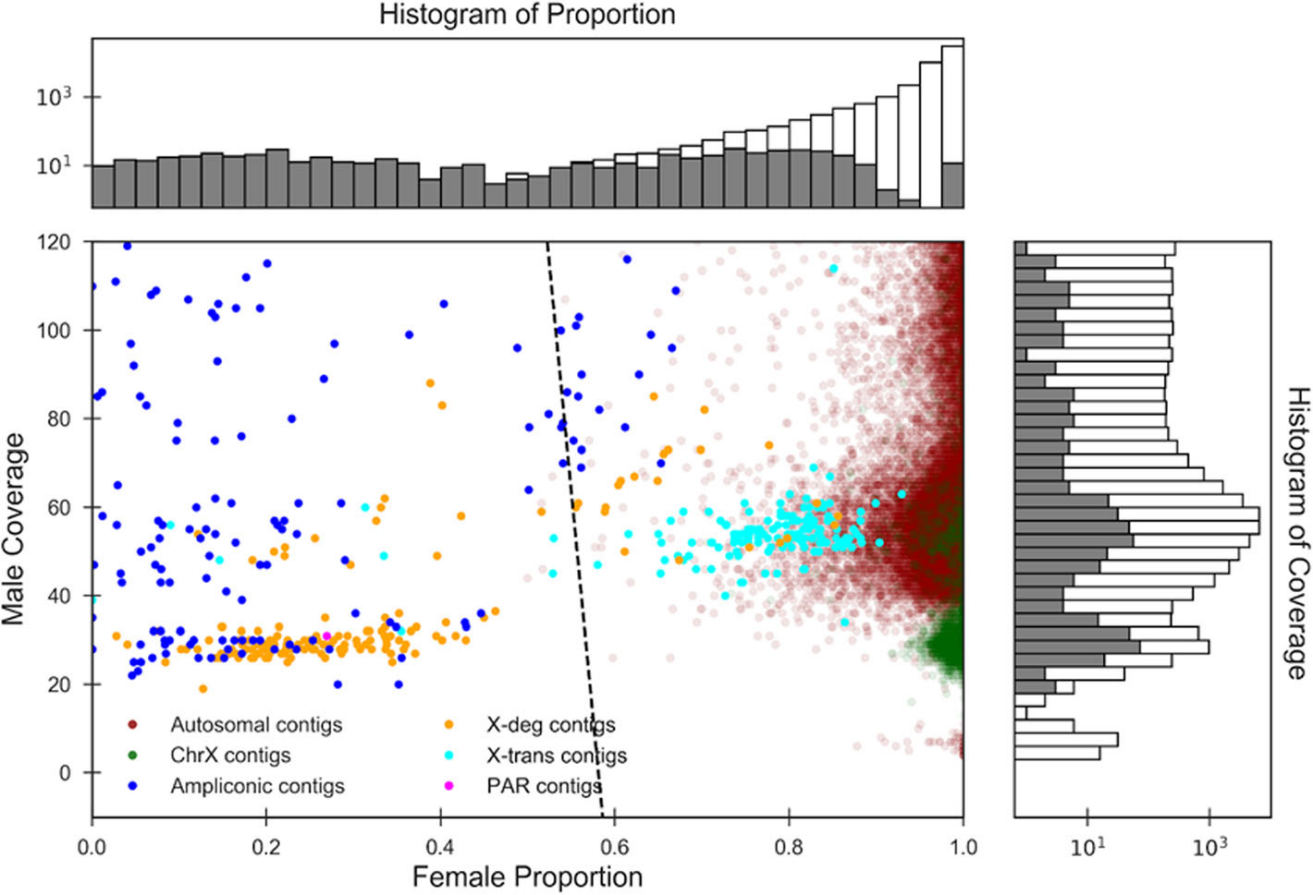
Scatter plot showing the proportion shared with female and male depth-of-coverage for each contig. This is the Illumina human male dataset with the hg38 female reference. Each point shows a single contig, with colors indicating the region of origin. Stacked histograms (log-scaled) on the top and right-hand side show the distribution of the female proportion and male depth of coverage values, respectively. The shaded regions of the histograms correspond to Y-contigs. Most of the PAR contigs have male depth of coverage about 120 and are hence not visible on this plot. The decision boundary output in best mode is shown as a dashed black line, with contigs to the left of the line classified as Y

In order to better understand the poor performance with respect to X-transposed sequences, Fig. 3 shows the scatter plot of female shared proportion vs. male depth of coverage for this assembly. The X-transposed contigs have a high female proportion, which is expected, since this region shares homology with the X chromosome. It may be possible to better recover X-transposed contigs and also improve PAR recovery if the female assembly has a labeled X chromosome; however, we do not pursue this approach here.

### Effect of technologies and reference qualities on DiscoverY

The results above rely on a high-quality female reference genome (i.e. hg38 after removing the Y chromosome), however, in most organisms, such a genome is not yet available. An alternative is to use a draft-quality female reference, which is available for many model organisms. However, draft-quality female assemblies are likely incomplete and the presence of gaps in such an assembly would result in a number of autosomal contigs having low proportion shared with the female; these might then be erroneously classified as Y-specific. This is best exemplified by the scatter plot of the 10X male and the draft PacBio female reference (Additional file 1: Figure S2a), which shows many such autosomal contigs.

We therefore compare the performance of DiscoverY on high- vs. draft-quality female references. The accuracy is certainly higher, e.g. the precision with a hg38 female is above 99.86%, regardless of the male data set, compared to at most 96.98% using PacBio or 10X female references (Fig. 1 and Additional file 1: Table S3). Taking the Illumina male dataset as an example, the precision and recall decrease by 5 and 9 percentage points, respectively, when switching from hg38 to a 10X female reference. The drop in recall happens across the board and does not affect any regions specifically (Fig. 2). The PacBio draft female assembly results in a much larger drop in quality, by 36 and 20 percentage points for precision and recall, respectively (Fig. 1). In terms of gene recovery, the 10X female reference is as good as hg38, but the PacBio reference does lead to fewer genes being recovered (Additional file 1: Figure S1).

Another consideration is that mixing different sequencing technologies for the female and male could adversely affect accuracy. With 10X, we do not observe this effect: fixing the female reference to be 10X, using a 10X male does not lead to a dominant accuracy compared to Illumina or PacBio males (Fig. 1). However, PacBio does seem to greatly benefit from being used for both the male and the female: fixing the female reference to be PacBio, using a PacBio male leads to markedly higher precision and recall compared with Illumina or 10X males (Fig. 1).

Finally, in the absence of any female reference, raw sequencing reads from a female can be used instead. This option gives higher accuracy compared to using the draft 10X or PacBio female references, when the female depth of coverage is at least 30x (Fig. 1). This effect may be due to the fact that low complexity or repetitive regions may be poorly assembled, leading to *k*-mers from those regions not being properly labeled as female. Raw sequencing data, however, would capture those *k*-mers. Thus, while investigating novel species for which a female reference does not exist, we recommend that a user start by generating low-coverage female sequencing data and run DiscoverY to check if its performance in retrieving Y-linked contigs matches expectations. If not, then more female reads might be necessary to improve the performance of this method; however, performing an assembly of the female data may be unnecessary.

### Effect of parameter selection

**Fig. 4:**
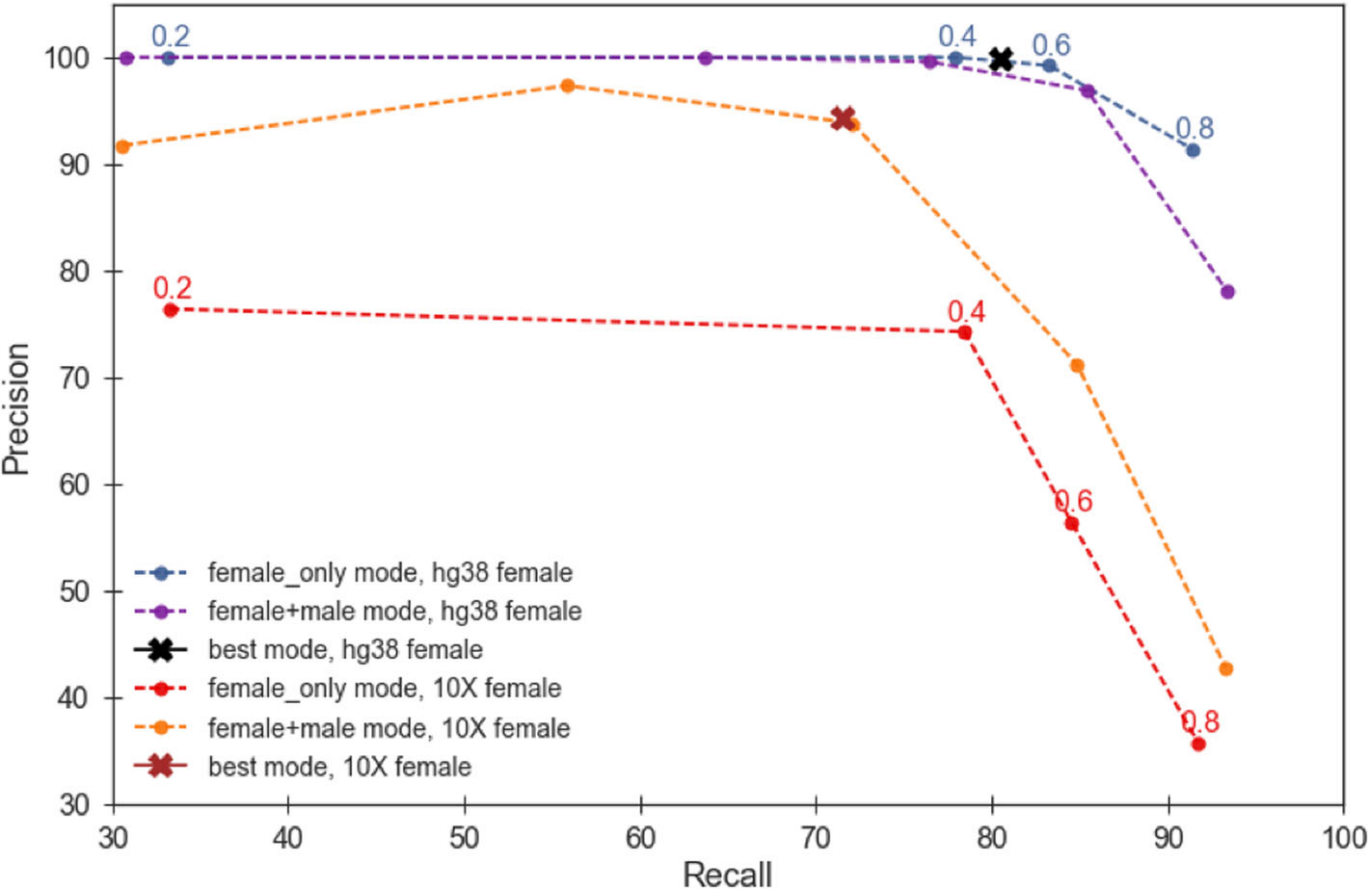
Effect of parameter settings on DiscoverY and comparison to YGS. DiscoverY was run across a broad range of parameter settings. Each curve reflects a combination of a given female reference genome (10X or hg38) and a mode (female+male or female_only); two points mark the results of best mode. The YGS method corresponds to the female_only mode. For the male, the Illumina dataset was used. The raw results, across the various parameters, are given in Additional file 1: Table S4; in this figure, the curve shows the upper perimeter of the convex hull of all the parameter settings (i.e. only those runs that produce the highest accuracy). These high-accuracy runs are identified as solid circles on the curve. In the case of female_only mode, the solid circles are labeled with the corresponding female proportion threshold

DiscoverY can produce plots of the type shown in Fig. 3, however, it is up to the user to decide what threshold to use to classify contigs as coming from the Y chromosome. These parameters represent a trade-off between precision and recall. To better understand this, we ran DiscoverY with a broad range of combinations of female proportion and male coverage threshold parameters. These results are shown in Fig. 4, with the raw numbers in Additional file 1: Table S4. The accuracy most heavily depends on the female proportion and less so on the male coverage threshold used (Additional file 1: Table S4). When it is possible to measure recall and precision, DiscoverY has an option to find the best linear separator. Figure 4 shows how this option performs; it obtains a reasonable compromise between precision and recall. However, we note that this is only useful when there exists a learning dataset from a related species with a similar quality of assembly.

### Comparing DiscoverY to existing methods

We first compared DiscoverY in female+male mode against the Chromosome Quotient (CQ) method, using the Illumina male reads and contigs and the Illumina female reads at 60x depth of coverage. In order to match the read length for the male and female datasets used by CQ, we used an Illumina female dataset that was from the same individual as the male contigs, but had read lengths of 151 bp (SRX847862-SRX848317). Scripts for the CQ method were downloaded from [17]. Figure 1 compares the performance of CQ (dashed grey curve) to DiscoverY (dashed black curve). CQ achieves higher levels of recall, but at a substantial drop in precision; DiscoverY, on the other hand, achieves higher precision (> 95%) compared to CQ (< 90%). In general, if the user has a target precision value that is > 80%, then DiscoverY achieves a strictly better recall than CQ.

Next, we compared DiscoverY in female+male mode to the Y Genome Scan (YGS) method. The YGS method is equivalent to the female_only mode, so this also amounts to a comparison of these two modes of DiscoverY. We performed two experiments and, for each experiment, ran DiscoverY in each of the two modes. We used Illumina male contigs and reads for both experiments, but we used a high-quality female reference (hg38-based) for one and a draft-quality female (10X) for the other. Figure 4 shows the results of comparing DiscoverY in female+male mode to the Y Genome Scan (YGS) method (raw numbers in Additional file 1: Table S4). When the reference quality is high (i.e. hg38), the YGS method performs comparably to DiscoverY in female+male mode. This is because proportion-only comparison to a high-quality female reference is sufficient to correctly reject putative false positives, and adding coverage information does not improve performance. However, when the reference is only of draft-quality (i.e. 10X), the YGS method performs poorly compared to DiscoverY in female+male mode. Looking at the scatter plot in Additional file 1: Figure S2b, we see that there is a large number of autosomal contigs with high male depth of coverage that do not share a high proportion with the female reference. These result in false positives identified by DiscoverY in female_only mode. However, male read coverage information can be used effectively in female+male mode to correctly reject these false positives.

### Time and memory requirements

Table 2 shows the runtime and memory usage of DiscoverY, measured on an x86_64 system with up to 64 available AMD Opteron 6276 processors and 512 GB available memory. Illumina male reads and contigs were used, together with a female hg38 reference. DiscoverY in female+male mode takes less than 14 h to run, plus an additional 3 h 20 m to construct the Bloom filter which stores all *k*-mers from the female reference. This is a one-time step; once the Bloom Filter has been computed, it is written to a file which can be rapidly loaded for any new male contigs. We have provided pre-computed female.bloom files for the hg38, 10x, and PacBio female references at [15]. DiscoverY uses 307 GB of RAM, due to Python’s heavyweight implementation of the male *k*-mer dictionary. This number can be reduced with an implementation in lower-level language like C++. In female_only mode, only 6 GB of RAM is needed, and the run time is under 8 h. The speed of DiscoverY is comparable to the CQ method, which completes in about 14 h.

**Table 2:** Runtime and memory usage of DiscoverY and CQ

	DiscoverY: female+male mode		DiscoverY: female_only mode		Chromosome Quotient	
Stage	Time	Max memory	Time	Max memory	Time	Max memory
Generating female *k*-mers Bloom Filter	3 h 20 m	6 Gb	3 h 20 m	6 Gb	N/A	N/A
Counting male *k*-mers abundance (DSK)	4 h 8 m	9 Gb	N/A	N/A	N/A	N/A
Computing proportion and coverage	9 h 2 m	307 Gb	3 h 49 m	5 Gb	N/A	N/A
Filtering of Y-contigs	5 m	< 1Gb	5 m	< 1Gb	N/A	N/A
Total	16 h 35 m	307 Gb	7 h 14 m	6 Gb	14 h 2 m	5 Gb

N/A: The CQ method is alignment-based and does not involve *k*-merization steps. Hence the corresponding fields of runtime and memory are left as “N/A”

### Applying DiscoverY to non-human samples

To test DiscoverY on non-human data, the latest build of gorilla female assembly (gorGor v5.0 [18]) was concatenated with the latest draft of the gorilla Y chromosome (gorY v1.0 [9]), to create a set of male contigs. As a result of this concatenation, no alignment was required to label the “true Y” contigs for validation. To have male depth of coverage info, whole-genome sequencing reads were generated from a male at 20x depth of coverage (see Methods). For female proportion calculation, using the same female reference from which we created the male contigs would overestimate DiscoverY’s performance. Therefore, whole-genome sequencing reads were generated from a female at a lower, 7x depth of coverage (see Methods); these reads were used in a place of a female reference.

DiscoverY achieved a precision of 92% and recall of 78%, similar to the performance on human data with similar depth of coverage female reads. For more details, Additional file 1: Figure S3 shows the scatter plot for the gorilla contigs. DiscoverY was also able to retrieve all X-degenerate genes from the Y chromosome (Additional file 1: Figure S1b), although not all genes were retrieved fully.

## Discussion and conclusion

We developed a method called DiscoverY for classifying Y-linked contigs from a whole-genome male assembly. DiscoverY leverages both a female genome (either a reference or a set of reads) and the coverage from the male reads used for assembly. When both the male contigs and the female reference is of high quality, DiscoverY has a precision of > 99.99% and a recall of 85%. Most of the false negatives are contigs from the X-transposed region and some from the pseudoautosomal (PAR) regions; however, other regions (e.g. ampliconic) can be retrieved with a recall of > 95%. We show that if a high-quality female reference is not available, a draft-quality reference can still be used to obtain good results; however, in some cases (e.g. the PacBio reference), the drop in quality is substantial. Alternatively, we show that reads from a female can be used in place of reference and can in fact lead to even higher accuracy than a draft-quality reference. Using different sequencing technologies for the male and female data was not detrimental except for the PacBio female, which only worked well with a PacBio male.

We show that DiscoverY compares favorably to two alternative approaches: the Y-genome scan and the Chromosome Quotient method. The Y-genome scan does not use depth of coverage from the male reads, and we show that when the female reference is not of high quality, such coverage information is important for improved accuracy. The Chromosome Quotient, on the other hand, uses coverage information from both male and female reads; however, it does not take advantage of a female reference when one is available.

DiscoverY can also potentially be used as “DiscoverW”, i.e. to retrieve female-specific W chromosome contigs from genomes with female heterogametic systems, for example in birds and butterflies. If used for this purpose, one needs to reverse all male notations to female notations (and vice versa) in this manuscript.

DiscoverY’s approach has limitations, in particular with respect to recall of regions with high homology to the X chromosome. These include the X-transposed and PAR regions. One possible way to overcome this is if the X chromosome is annotated in the female reference, in which case these contigs could potentially be identified as (1) having shared *k*-mers with chromosome X but not the autosomes and (2) having twice the depth of coverage of other such contigs. Alternatively, DiscoverY could be combined with flow-sorting or other enrichment techniques to increase the proportion of Y chromosome prior to sequencing. By doing so, the PAR and X-transposed regions will show up as having much higher depth of coverage than autosomal or X-contigs. We leave these directions as future work.

Further, using DiscoverY with just one individual might lead to mis-classification of autosomal contigs coming from regions that are deleted in the female assembly being compared against. This can be ameliorated by running DiscoverY on multiple dataset pairs separately and then merging the results by only retaining putative Y sequences if they are shared across different runs. However, we also note that in some cases, such as the great apes, obtaining multiple samples can be problematic.

Additionally, given that contigs may be mosaics of different sequence classes, relying on coverage might mis-classify contigs that span the boundary of a single copy region (such as an X-degenerate region) and a multicopy region (such as an ampliconic region). We might also expect to see this at the boundary of evolutionary strata on X-degenerate regions. However, since there are only a handful of such boundaries or sequence class transitions, we expect only a small number of such instances where a contig maybe be composed of a mosaic of sequence classes with highly differing copy numbers. The DiscoverY method is also inured to this due to the choice of median instead of mean coverage, which is less sensitive to outliers that can skew a contig’s coverage—for example, if some parts of the contig is composed of repetitive regions.

In evaluating the performance of DiscoverY, we note that the validation may not properly address novel sequence insertions. As the precision is measured with respect to the hg38 reference - any novel Y sequence in the NA24385 individual that is correctly classified as Y would mistakenly count against the precision. Since the proportion of total novel sequence in one individual is likely to be small, we do not expect this to have a large effect. Moreover, in comparing different tools and technologies, the effect will manifest itself only in the case that some tools or technologies are able to classify novel insertions better than others. In terms of computational requirements, DiscoverY in its male+female mode is memory- intensive - although the memory footprint can be reduced by an order of magnitude by running DiscoverY in female-only mode.

Finally, we are working on extending DiscoverY to not only classify contigs from a male assembly but also long male reads from Oxford Nanopore Technology. Given the length of these reads, there might be enough signal to do a filtering step prior to assembly, thus speeding up and simplifying the assembly process.

## Methods

### DiscoverY

**Fig. 5:**
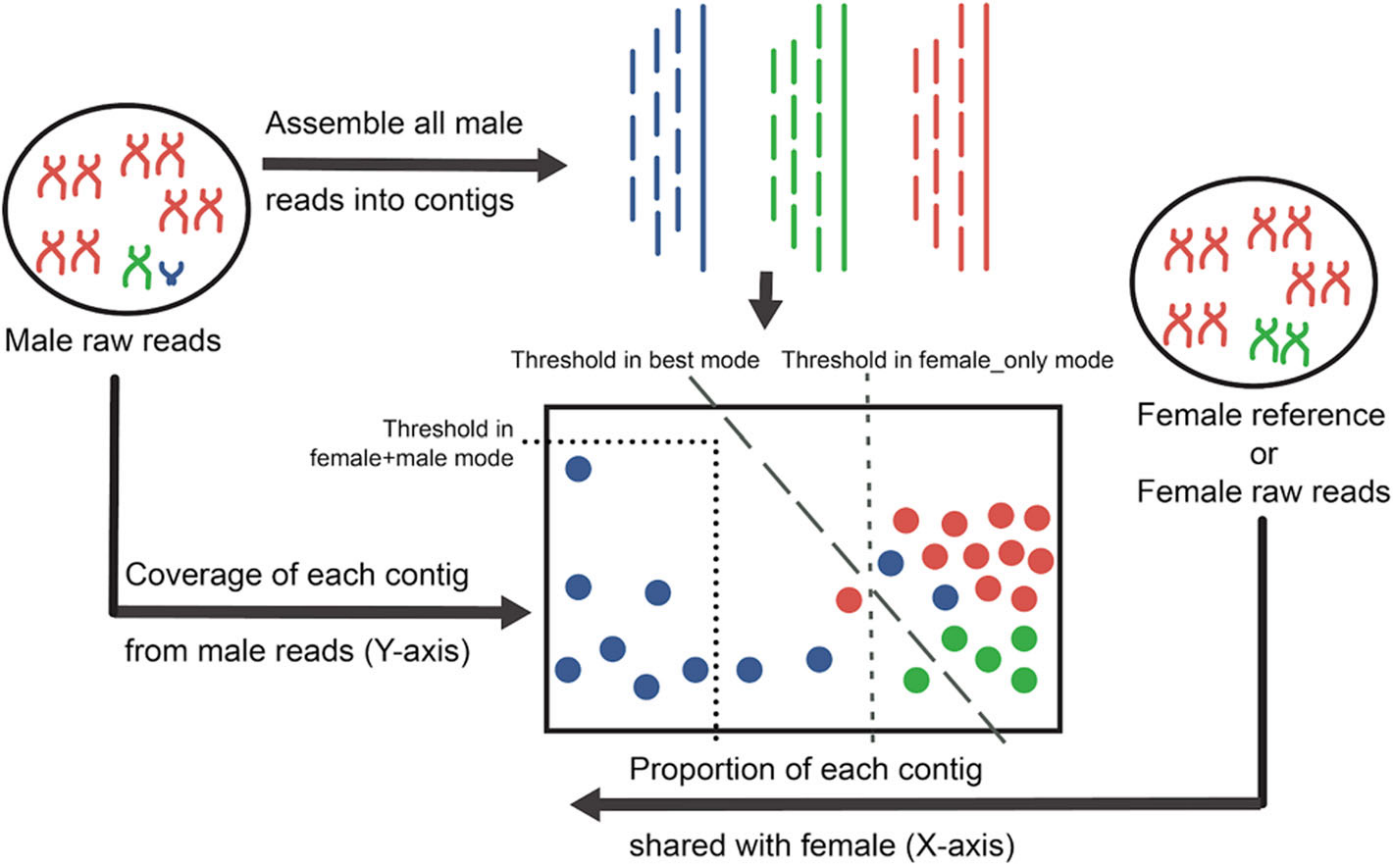
Overview of DiscoverY. A male genome is sequenced and assembled into contigs, which are colored red (autosomes), green (X chromosome), and blue (Y chromosome). In parallel, a female reference is *k*-merized and proportion shared with female is computed for each contig (female proportion). Additionally, the median *k*-mer abundance for each contig is calculated based on the male raw reads used for assembly (male depth of coverage). The vertical line (short dashes) indicates a threshold chosen in female_only mode, using only the female proportion. In this mode, those contigs with low proportion shared with female reference are classified as Y-chromosomal. The diagonal line (long dashes) indicates a threshold chosen in best mode, using both female proportion and male coverage. In this mode, those contigs with low-to-moderate proportion shared with female reference and low depth of coverage from male read *k*-mers are classified as Y- chromosomal. The pair of dotted lines indicates a threshold chosen in female+male mode. In this mode, the user can manually try different combinations of female proportion and male coverage to retrieve Y chromosomal contigs

As an input to DiscoverY, the user provides contigs from a male assembly, raw reads used to generate those contigs (that we refer to as male reads), and a female reference (or reads) to compare against (Fig. 5 shows the schematic workflow for DiscoverY). For every contig in the male assembly, DiscoverY then calculates and outputs two features: the proportion shared with female, and depth of coverage from male reads. To compute the proportion shared with female, exact *k*-mer matching is used to compare male contigs to a female reference or to female raw sequencing reads. As a first step, the female reference (or raw sequencing reads) is decomposed into all overlapping substrings of size *k* (*k-*mers). To speed up the *k*-mer decomposition process in the case of high-depth sequencing of female raw reads, the *k*-mer counter DSK is used with default parameters [19], and *k*-mers that occur fewer than three times in the dataset are filtered out, as these are likely erroneous *k*-mers. Next, the female *k-*mer set is stored in a Bloom filter for low-memory usage and efficient retrieval using the python library pybloomfiltermmap [20]. Subsequently, each male contig is decomposed into its constituent *k*-mers, and the female Bloom Filter is queried for each of these *k-*mers. The ratio of the number of successful lookups in the female set to the total number of *k-*mers from that contig is recorded for each contig as *proportion shared with female*.

To compute the male depth of coverage of each contig, the male reads used for assembly are *k*-merized and the abundance of each *k*-mer in the read dataset is recorded using DSK. DSK is run with default parameters, after filtering out *k*-mers that occur fewer than three times in the dataset. As before, each male contig is then decomposed into its constituent *k*-mers, and the median abundance across all *k*-mers in a contig is recorded. This median *k*-mer abundance for every contig serves as the contig’s *depth of coverage from male reads*.

After computing these two features, DiscoverY generates a scatter plot where every point is a contig and the axes are the two features—proportion shared with female and depth of coverage from male reads (a schematic is shown in Fig. 5 and a real example in Fig. 3). The user can manually inspect this plot and decide on the best approach to classify which contigs originated from the Y chromosome. DiscoverY implements three natural ways to perform the classification, as described below, but the user can in principle use any alternate approaches.

In female_only mode, the user can specify a female proportion threshold below which the contig is classified as Y-chromosomal. This mode is useful in the case where the reads used to generate the male contigs are not available, and, hence, male coverage cannot be computed. This method can be considered as a Python implementation of the YGS.pl script available with the Y Genome Scan method [12]. Figure 5 shows a schematic of expected proportion for different contigs, which are represented as points on this plot. Most Y-chromosomal contigs are expected to have low female proportion (due to male specificity), except for those contigs from X-transposed and PAR regions, or contigs consisting of repeats shared between the Y and other chromosomes. For example, the X-transposed regions of the Y on the human share anywhere between 60 to 90% of their *k-*mers with the female. Non-Y-chromosomal contigs are expected to have high female proportion. The value of the female proportion threshold thus controls a trade-off between precision and recall, where increasing the threshold can help improve sensitivity.

In female+male mode, the user can specify a threshold for female proportion and a threshold for male coverage. If a contig falls below both thresholds, it is classified as Y-chromosomal. Adding an upper bound on male depth of coverage can help filter out autosomal repeats that are not properly captured in the female reference. Note that we do not suggest a male_only mode because using only low coverage from male reads to inform Y-contig selection would result in a loss of regions occurring in multiple copies on the Y (especially ampliconic regions which are male-specific, but found in multiple copies).

Another approach is to draw a line on the plot, i.e. a linear separator. DiscoverY uses this approach when in best mode. The idea of this mode is that in certain scenarios, the user wants to run DiscoverY on a training dataset which has a labeled ground truth, i.e. each contig is already labeled according to whether or not it comes from the Y chromosome. For example, a training dataset may be obtained by targeted sequencing of conserved genes on the Y (e.g. X-degenerate genes in the gorilla, such as in [21]. DiscoverY can then learn the best-fit linear separator for this dataset, so that the parameters can be applied to another dataset which is expected to have similar properties (e.g. whole-genome sequencing of the gorilla). To find the best fit, DiscoverY uses Linear Support Vector Classification [22], as implemented in the LinearSVC function from the python library sklearn.svm (sklearn version 0.18.1). In order to reduce the chance of overfitting the training set, the contigs were randomly partitioned into a training set (16%), test set (4%), and the validation set (80%).

In our experiments, we set the *k*-mer size *k* = 25. Previous experiments with Y chromosome sequence isolation tools such as RecoverY [10] suggested that *k*-mer size of 25 performs well for calculating *k*-mer coverage in mammalian Y chromosomes from sequencing data.

### Experimental methods

To test DiscoverY on non-human data, gorilla male genomic DNA (ID KB3781) was extracted from a fibroblast cell line provided by the San Diego Zoological Society. An Illumina paired-end library was constructed using the TruSeq DNA Sample Preparation Kit. Two Illumina mate pair libraries were constructed from male genomic DNA (applying a narrow 7–8 kb and a broad 5–10 kb BluePippin DNA size selection) using Nextera Mate Pair Library Preparation Kit. All libraries were sequenced (2 × 151 bp) on the HiSeq2500 (Rapid mode) and concatenated together to provide a gorilla male dataset at ~20x depth of coverage for male coverage calculation. Gorilla female reads at low depth of coverage (7x) were also generated from an unrelated female. Gorilla female genomic DNA (ID 2000–0150) was isolated from liver provided by the Smithsonian Institution with the DNeasy Blood and Tissue kit (Qiagen). An Illumina paired-end library was constructed using the TruSeq DNA Sample Preparation Kit, and the library was sequenced (2 × 151 bp) on the HiSeq 2500 (Rapid mode).

### Dataset processing

We used several existing male assemblies for analysis, as summarized in Table 1 and Additional file 1: Table S1. For 10X, we used the ‘raw’ assembly output generated by Supernova [23]. The 10X raw assembly represents every edge of the assembly graph as a FASTA record, including cycles, microbubble arms and gaps; this captures difficult-to-sequence regions such as those found on the Y chromosome. For the PacBio assembly, we used error-corrected reads, which is necessary due to PacBio’s high error rate. For the Illumina and PacBio male assemblies, contigs of length less than 1000 bp were discarded. For the larger, more fragmented 10X raw assembly, we discarded contigs shorter than 10 kb, as done by the authors of that assembly [23].

### Evaluation criteria

For the purposes of evaluation, we labeled each of the male contigs with a “true” chromosome as follows. Each contig was mapped to the hg38 male reference using the minimap2 aligner [24] version 0.2 with parameters -x asm5 --secondary = no. Multi-mappings (in which a single contig mapped to multiple chromosomes) were resolved in two steps. First, any alignment with mapping fraction (i.e. the alignment length divided by total query length) less than 50% was discarded. Subsequently, if a multi-mapping still existed for a contig, the alignment with highest alignment identity (i.e. the number of matches divided by total alignment length) was selected. Contigs mapping to the Y chromosome were further classified if they came from one of the four annotated regions on the Y chromosome: X-degenerate, Ampliconic, X-Transposed and PAR; this was done based on the contig alignment locations (Additional file 1: Table S2).

To measure the accuracy of DiscoverY, we used precision and recall. Let *x* be the total length of contigs that were both chosen by DiscoverY and marked “true Y” according to alignment. Precision is defined as *x* divided by the length of all contigs chosen by DiscoverY. Recall is defined as *x* divided by the length of all contigs marked as “true Y”.

## Additional file

Additional file 1: Supplementary figures and tables. (PDF 1263 kb)

## Data Availability

The datasets generated and/or analyzed during the current study has been submitted to the NCBI BioProject [25] as BioProject PRJNA555244. DiscoverY is freely available at [16].

## Abbreviations

CQ: Chromosome Quotient
PacBio: Pacific Biosciences
PAR: Pseudoautosomal Region
SHIMS: Single-Haplotype Iterative Mapping and Sequencing
SMRT: Single Molecule Real-Time
YGS: Y-Genome Scan
