# Supplementary material for: DiscoverY: a classifier for identifying Y chromosome sequences in male assemblies
Source: BMC Genomics. 2019 Aug 9;20:641. doi: 10.1186/s12864-019-5996-3 (PMC6688218; doi:10.1186/s12864-019-5996-3)
Supplement: Supplementary file 1 — Supplementary figures and tables. (PDF 1263 kb) [file 12864_2019_5996_MOESM1_ESM.pdf]

Supplementary Material

DiscoverY: A classifier for identifying Y chromosome sequences in male assemblies

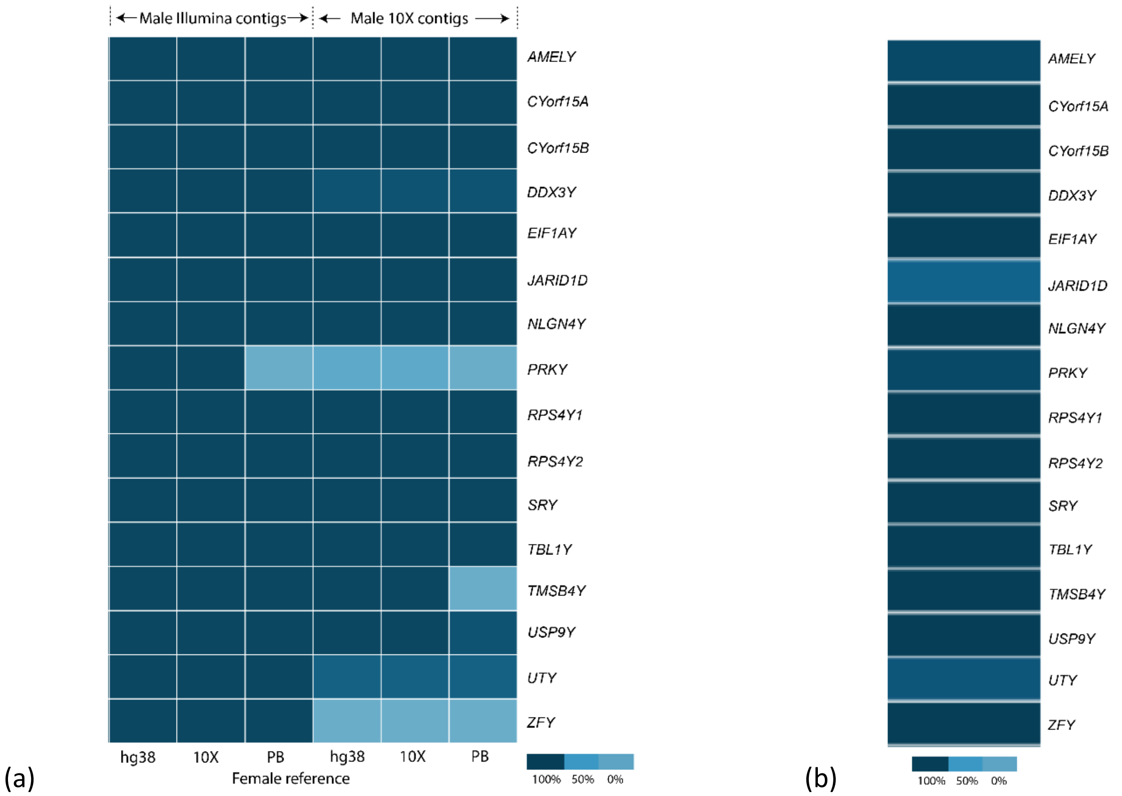

Figure S1. Retrieval of X-degenerate genes. (a) To check for the retrieval of X-degenerate genes by DiscoverY assemblies, sequences of 16 single copy human X-degenerate genes were mapped to the assemblies using BWA mem (version 0.7.5a-r428) [1] with seed length = 5 to increase sensitivity. The percentage in the heat map is the length of the gene sequence mapping to the assembly. The assemblies shown in the left heatmap are for the human Illumina male using the female reference as hg38 (column 1), 10X (column 2), and PacBio (column 3). (**b**) The right panel shows the heatmap for the gorilla contigs

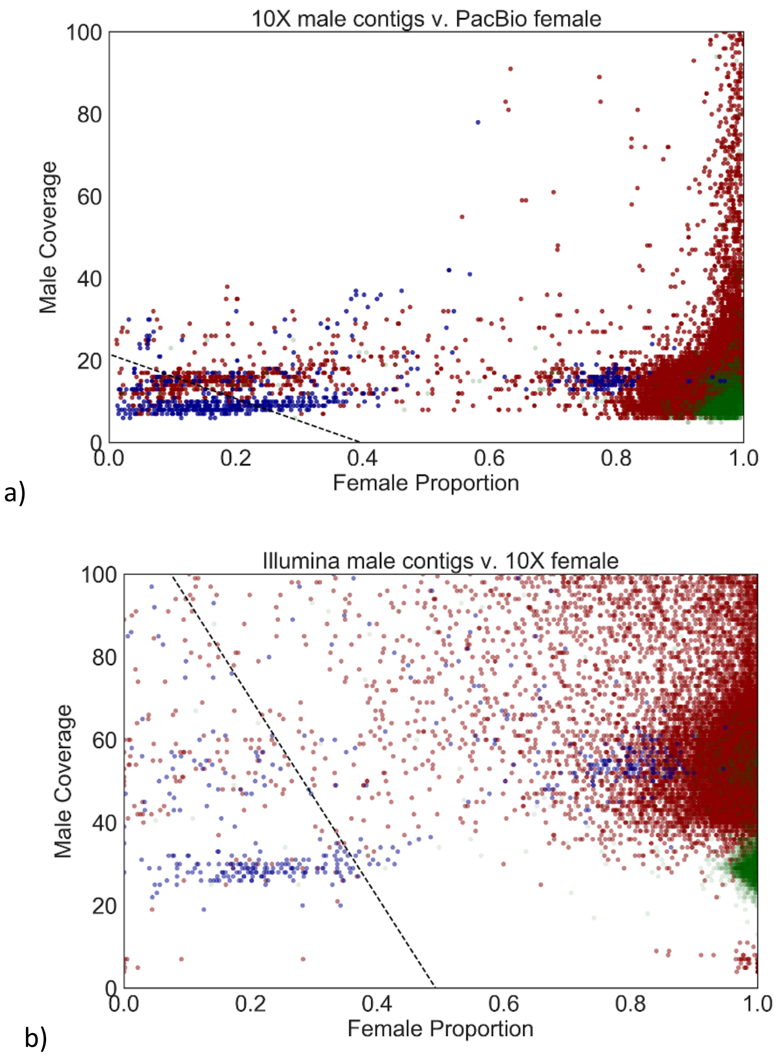

Figure S2. Scatter plot for the 10X male and PacBio female run (panel a) and the Illumina male and 10X female run (panel b). Y-chromosomal contigs are represented in blue, autosomal contigs in red, and X-chromosomal contigs in green.

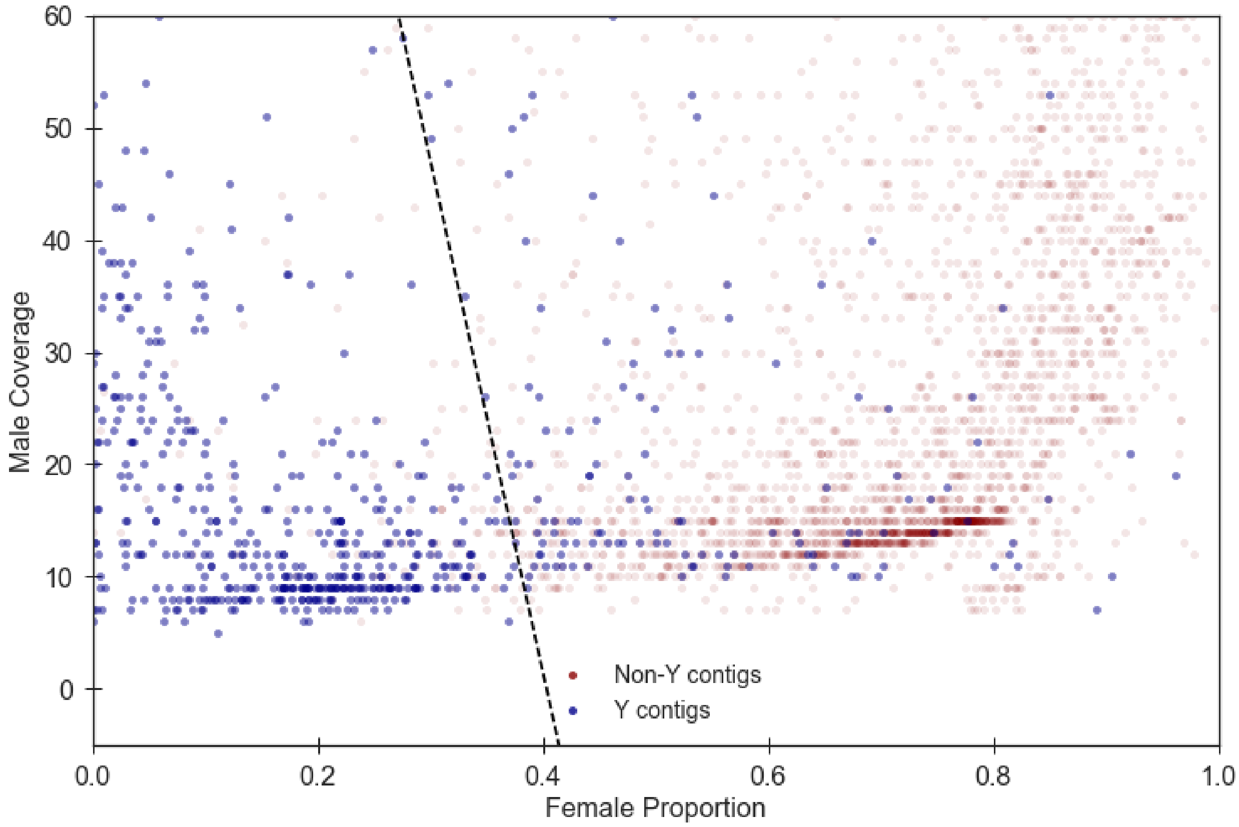

Figure S3. Gorilla contigs plotted with female proportion and male coverage. Among the Y chromosomal contigs to the left, note a dense cluster at ~8x coverage. Since this is half the depth of coverage of autosomal contigs (~16x), these contigs are likely from single-copy regions on the Y chromosome.

Table S1. Accession numbers of datasets used to test DiscoverY.

Dataset				Accession	Accession	
**Org**	**Tech**	**Sex**	**Sample**	**for Reads**	**for Contigs**	**Citation**
Human	Illumina	M	NA24385	GIAB ftp site [2]	GIAB ftp site [3]	[4]
Human	10X	M	NA24385	SRX1392293-SRX1392296	GCA_002023025.1	[5]
Human	PacBio	M	NA24385	SRX1033793–SRX1033798	GCA_001542345.1	[4], [6]
Human	Illumina	F	NA12878	SRX1049768-SRX1049855	N/A	[4]
Human	mixed	F	hg38	N/A	GCA_000001405.15	[7]
Human	10X	F	NA12878	N/A	GCA_002022845.1	[5]
Human	PacBio	F	NA12878	N/A	GCA_001013985.1	[8]
Gorilla	Illumina	M	see Methods	SRX6461472-SRX6461474	N/A	this paper
Gorilla	Illumina	F	Gg6	SRX6461471	N/A	this paper

Table S2. Region-by-region breakdown of the human Y chromosome. Note that some regions occur as a series of interspersed tracts.

Sequence class of Y	Start coordinate on hg38 (Mb)	End coordinate on hg38 (Mb)	Total Length (Mb)
PAR (Total)	***N/A***	***N/A***	*4.0*
PAR 1	0	2.8	2.8
PAR 2	56.5	57.7	1.2
X-transposed (Total)	*3.1*	*6.5*	*3.4*
X-degenerate (Total)	***N/A***	***N/A***	*10.5*
X-degenerate (Tract 1)	2.8	3.1	0.3
X-degenerate (Tract 2)	6.5	7.5	1.0
X-degenerate (Tract 3)	10.6	15.8	5.2
X-degenerate (Tract 4)	16.2	17.5	1.3
X-degenerate (Tract 5)	18.8	21.5	2.7
Ampliconic (Total)	***N/A***	***N/A***	*10.0*
Ampliconic (Tract 1)	7.5	10.3	2.8
Ampliconic (Tract 2)	15.8	16.2	0.4
Ampliconic (Tract 3)	17.5	18.8	1.3
Ampliconic (Tract 3)	21.5	27.0	5.5
Centromere (Total)	*10.3*	*10.6*	*0.3*

Table S3. Accuracy of DiscoverY on different datasets (raw numbers for Figure 3).

Male Dataset	Female data	True Y by Mapping (Mb)	"Y" according to DiscoverY (Mb)	Precision	Recall	Female Proportion Threshold	Male Coverage Threshold
Illumina	hg38	17.58	14.17	99.86	80.51	0.58	1186
Illumina	10X	17.58	13.32	94.36	71.49	0.49	117
Illumina	PacBio	17.58	16.79	63.27	60.43	0.35	143
10X	hg38	24.44	19.98	99.99	81.78	0.64	484
10X	10X	24.44	21.97	89.56	80.52	0.54	223
10X	PacBio	24.44	18.53	62.11	47.1	0.39	21
PacBio	hg38	18.09	13.71	99.91	75.74	0.51	428
PacBio	10X	18.09	13.66	96.98	73.24	0.5	114
PacBio	PacBio	18.09	13.44	97.32	72.34	0.43	109
gorGor 5.0 + gorY v1.0	Gorilla Illumina	25.35	21.55	92.68	78.81	0.4	183
Illumina	Illum. reads at 7.5x	17.58	15.03	89.64	76.65	0.38	707
Illumina	Illum. reads at 15x	17.58	15.1	92.76	79.67	0.49	2792
Illumina	Illum. reads at 30x	17.58	14.78	97.41	81.93	0.53	1566
Illumina	Illum. reads at 60x	17.58	15.14	97.11	83.65	0.57	1306
Illumina	Illum. reads at 120x	17.58	14.89	97.26	82.38	0.58	3131

Table S4. Effect of parameters on DiscoverY (raw numbers used to generate Figure 5). The male dataset used is Illumina. The runs used as points for the convex hull in Figure 5 are shown in bold.

Female Data	Mode	True Y by Mapping (Mb)	"Y" according to DiscoverY (Mb)	Precision	Recall	Female Proportion Threshold	Male Coverage Threshold
**hg38**	**female+male**	**17.58**	**5.39**	**100**	**30.7**	**0.2**	**80**
hg38	female+male	17.58	8.48	100	48.23	0.3	30
hg38	female+male	17.58	9.12	100	51.89	0.3	40
hg38	female+male	17.58	9.65	100	54.93	0.3	50
hg38	female+male	17.58	10.67	100	60.68	0.3	60
hg38	female+male	17.58	10.8	100	61.42	0.3	70
hg38	female+male	17.58	11.11	100	63.22	0.3	80
hg38	female+male	17.58	11.18	100	63.59	0.3	90
hg38	female+male	17.58	11.24	100	63.96	0.3	100
hg38	female+male	17.58	10.98	100	62.49	0.4	40
hg38	female+male	17.58	12.66	100	72.05	0.4	60
hg38	female+male	17.58	11.81	99.97	67.19	0.5	50
**hg38**	**female+male**	**17.58**	**11.19**	**99.98**	**63.64**	**0.6**	**40**
hg38	female+male	17.58	12.96	99.88	73.63	0.6	60
hg38	female+male	17.58	9.82	99.97	55.85	0.7	30
**hg38**	**female+male**	**17.58**	**13.49**	**99.59**	**76.43**	**0.7**	**70**
hg38	female+male	17.58	9.82	99.92	55.85	0.8	30
hg38	female+male	17.58	11.24	99.53	63.65	0.8	40
hg38	female+male	17.58	12.22	98.13	68.23	0.8	50
hg38	female+male	17.58	14.11	97.75	78.5	0.8	60

hg38	female+male	17.58	14.57	97.2	80.62	0.8	70
hg38	female+male	17.58	15.05	97.17	83.21	0.8	80
hg38	female+male	17.58	15.35	97.07	84.78	0.8	90
**hg38**	**female+male**	**17.58**	**15.5**	**96.87**	**85.43**	**0.8**	**100**
hg38	female+male	17.58	9.83	99.86	55.85	0.9	30
hg38	female+male	17.58	11.51	97.19	63.66	0.9	40
hg38	female+male	17.58	13.54	88.91	68.48	0.9	50
hg38	female+male	17.58	18.69	80.83	85.92	0.9	60
hg38	female+male	17.58	19.74	78.88	88.52	0.9	70
hg38	female+male	17.58	20.32	78.82	91.11	0.9	80
hg38	female+male	17.58	20.69	78.73	92.68	0.9	90
**hg38**	**female+male**	**17.58**	**20.99**	**78.15**	**93.33**	**0.9**	**100**
hg38	best	17.58	14.17	99.86	80.51	auto	auto
**hg38**	**female_only(YGS)**	**17.58**	**5.83**	**100**	**33.16**	**0.2**	**n/a**
**hg38**	**female_only(YGS)**	**17.58**	**13.7**	**100**	**77.94**	**0.4**	**n/a**
**hg38**	**female_only(YGS)**	**17.58**	**14.75**	**99.22**	**83.26**	**0.6**	**n/a**
**hg38**	**female_only(YGS)**	**17.58**	**17.57**	**91.42**	**91.39**	**0.8**	**n/a**
**10X**	**female+male**	**17.58**	**5.86**	**91.76**	**30.59**	**0.2**	**80**
10X	female+male	17.58	8.51	97.8	47.39	0.3	30
10X	female+male	17.58	9.3	96.46	51.04	0.3	40
10X	female+male	17.58	9.98	95.25	54.09	0.3	50
10X	female+male	17.58	11.19	94	59.84	0.3	60
10X	female+male	17.58	11.37	93.67	60.58	0.3	70

10X	female+male	17.58	11.69	93.73	62.37	0.3	80
10X	female+male	17.58	11.8	93.45	62.75	0.3	90
10X	female+male	17.58	11.9	93.2	63.12	0.3	100
10X	female+male	17.58	11.4	96.31	62.49	0.4	40
**10X**	**female+male**	**17.58**	**13.5**	**93.86**	**72.08**	**0.4**	**60**
10X	female+male	17.58	12.48	94.64	67.19	0.5	50
10X	female+male	17.58	11.71	95.51	63.64	0.6	40
10X	female+male	17.58	14.13	91.55	73.6	0.6	60
**10X**	**female+male**	**17.58**	**10.03**	**97.87**	**55.85**	**0.7**	**30**
10X	female+male	17.58	15.66	85.78	76.4	0.7	70
10X	female+male	17.58	10.08	97.33	55.85	0.8	30
10X	female+male	17.58	12.17	91.94	63.65	0.8	40
10X	female+male	17.58	11.99	85.36	68.23	0.8	50
10X	female+male	17.58	17.09	80.79	78.56	0.8	60
10X	female+male	17.58	18.57	76.38	80.68	0.8	70
10X	female+male	17.58	19.8	73.94	83.27	0.8	80
**10X**	**female+male**	**17.58**	**20.94**	**71.22**	**84.84**	**0.8**	**90**
10X	female+male	17.58	22.79	65.94	85.49	0.8	100
10X	female+male	17.58	10.28	95.47	55.85	0.9	30
10X	female+male	17.58	12.96	86.31	63.66	0.9	40
10X	female+male	17.58	17.05	70.61	68.48	0.9	50
10X	female+male	17.58	26.58	56.81	85.9	0.9	60
10X	female+male	17.58	30.15	51.6	88.5	0.9	70
10X	female+male	17.58	32.32	49.54	91.08	0.9	80

10X	female+male	17.58	35	46.54	92.66	0.9	90
**10X**	**female+male**	**17.58**	**38.37**	**42.74**	**93.3**	**0.9**	**100**
10X	best	17.58	13.32	94.36	71.49	auto	auto
**10X**	**female_only(YGS)**	**17.58**	**7.65**	**76.4**	**33.24**	**0.2**	**n/a**
**10X**	**female_only(YGS)**	**17.58**	**18.57**	**74.25**	**78.45**	**0.4**	**n/a**
**10X**	**female_only(YGS)**	**17.58**	**26.34**	**56.41**	**84.52**	**0.6**	**n/a**
**10X**	**female_only(YGS)**	**17.58**	**45.11**	**35.74**	**91.71**	**0.8**	**n/a**

References

[1] Li H, Durbin R. 2009. Fast and accurate short read alignment with Burrows– Wheeler transform. Bioinformatics 25: 1754–1760.

[2] Genome In A Bottle FTP site. ftp://ftp-trace.ncbi.nlm.nih.gov/giab/ftp/data/AshkenazimTrio/HG002_NA24385_son/NIST_Illumina_2x250bps/novoalign_bams/HG002.GRCh38.2x250.bam. Accessed 18 June 2018.

[3] Genome In A Bottle FTP site. ftp://ftp-trace.ncbi.nlm.nih.gov/giab/ftp/data/AshkenazimTrio/analysis/TAMU_NIST_Illumina_2x250bps_DISCOVAR_Assemblies_09162016/son. Accessed 18 June 2018.

[4] Zook, J.M. et al. Extensive sequencing of seven human genomes to characterize benchmark reference materials. Sci. Data 3, 160025 (2016).

[5] Weisenfeld N. et al. 2017. "Direct Determination of Diploid Genome Sequences." Genome Research 27 (5): 757–67.

[6] Berlin K, et al. 2015. Assembling large genomes with single-molecule sequencing and locality-sensitive hashing. Nat Biotechnol 33: 623–630

[7] Schneider, V.A. et al. Evaluation of GRCh38 and de novo haploid genome assemblies demonstrates the enduring quality of the reference assembly. Genome Res. 27, 849–864 (2017).

[8] Pendleton, M. et al. Assembly and diploid architecture of an individual human genome via single-molecule technologies. Nat. Methods 12, 780–786 (2015)
